# Total Hip Arthroplasty Learning Curves Based on Approach for New Fellowship-trained Surgeons

**DOI:** 10.5435/JAAOSGlobal-D-23-00094

**Published:** 2023-07-06

**Authors:** James C. Messina, Justin A. Magnuson, Christopher M. Melnic, Nicholas B. Frisch, Chad A. Krueger, Matthew J. Grosso

**Affiliations:** From the Department of Orthopaedic Surgery, University of Connecticut, Farmington, CT (Dr. Messina); Rothman Orthopaedic Institute, Thomas Jefferson University, Philadelphia, PA (Dr. Magnuson and Dr. Krueger); the Department of Orthopaedic Surgery, Massachusetts General Hospital, Boston, MA (Dr. Melnic); the Department of Orthopedic Surgery, Ascension Providence Rochester, Rochester, MI (Dr. Frisch); and Connecticut Joint Replacement Institute, Hartford, CT (Dr. Grosso).

## Abstract

There is concern that the direct anterior (DA) approach is associated with a steeper learning curve than the posterolateral (PL) approach in total hip arthroplasty (THA). The purpose of this study was to investigate if the learning curve is similar for newly trained arthroplasty fellowship-trained surgeons using the DA and PL approaches.

**Methods:**

The first 100 primary THA cases of six DA and PL fellowship-trained arthroplasty surgeons were identified and divided into 50 case cohorts. Demographics, indications for surgery, and 90-day Hip Society standardized complications were collected. Variables were analyzed using independent sample *t* tests, chi-square tests, or Fisher exact tests.

**Results:**

In total, there were 600 patients, and no notable differences were observed in revision surgeries, surgical complications, and total complications between the DA and PL groups. Both groups had lower revision surgery rates, surgical complications, and total complications during their second 50 cases. Higher revision surgeries, and surgical and total complication rates were observed among all surgeons during the first 50 cases.

**Discussion and Conclusion:**

No differences were observed in the learning curve when comparing the DA and PL approach. With proper training, early-career surgeons can safely perform THA with similar complication rates regardless of the approach.

The posterolateral (PL) approach to the hip is currently the most common approach for total hip arthroplasty (THA) in the United States.^1^ However, the direct anterior (DA) approach continues to show increased usage among surgeons in practice, with increasing prevalence in resident/fellow education, patient preference, and surgeon marketability.^2-4^ A recent survey in 2020 noted that the DA approach accounted for 45% of primary THA, an increase in 5% since 2018.^1,5^ Much of this increased popularity stems from potential, although still debated, benefits of reduced soft-tissue damage, decreased risk of dislocation, quicker recovery times, and earlier return to activities in the early postoperative period.^6,7^

Despite this increased popularity, like many newly adopted surgical techniques, the DA approach has been associated with higher complication rates among its new users. Several studies have demonstrated higher rates of wound complications, intraoperative fracture, neurapraxia, and revisions when comparing DA with other approaches.^8-12^ These studies suggest a notable learning curve for the technique before complication rates return to a lower level. As an example, the Australian Orthopaedic Association National Joint Replacement Registry Annual Report found a higher rate of revision and early fracture of DA approach compared with the PL and lateral approaches.^13^ Given these findings, there has been concern that the DA approach is associated with a much steeper learning curve than the PL approach.

The ‘‘learning curve,” in the context of surgeons, has generally referred to the number of cases needed to achieve a steady state of outcomes.^9^ This has been established by measuring operation times or procedure-related complication rates. Training, experience, ability, and surgical team may all influence these measures. Previous studies have attempted to quantify this learning curve for DA and have found this learning curve to be anywhere from 40 to 100 cases.^12,14-18^ The most studies evaluated surgeons transitioning to an anterior-based approach after having had already been trained in a different approach such as PL. As a result, there is a paucity of literature on learning curves of fellowship-trained DA surgeons during their first year of practice as compared with other approaches. In addition, there are no studies that compare the learning curve based on the approach for fellowship-trained arthroplasty surgeons.

The goal of this study was to investigate if the learning curve is similar for newly fellowship-trained arthroplasty surgeons beginning independent practice primarily using the DA approach compared with surgeons using PL approaches regarding their complications. We hypothesized that there would be no difference between the two groups.

## Methods

Before conducting the study, Institutional Review Board approval at the corresponding institutions was obtained. The first 100 cases of six recently graduated fellowship-trained arthroplasty surgeons (three DA surgeons, three PL surgeons) were evaluated. The surgeries were done between January 2018 and January 2021.

The DA surgeons were fellowship trained at one institution and extensively used a DA approach for primary THA. The DA surgeons in this study perform the surgery without a specialized table or intraoperative fluoroscopy. The PL surgeons were also fellowship trained at one institution and extensively used a PL approach for primary THA. Inclusion criteria included the first 100 patients of each fellowship-trained arthroplasty surgeon during their first year in practice undergoing primary THA. The surgeons exclusively used the respective approach emphasized in their fellowship for their first 100 cases. No crossover of approach to PL was found for complex primary THA among the DA group. All nonprimary THA, including conversion THA, was excluded from the study.

The first 100 primary THA cases in practice were collected and divided into cases 1 to 50 and 51 to 100 for each surgeon. Age, sex, body mass index (BMI), and laterality of surgery were collected for each patient along with the primary indication for surgery. Medical records were reviewed to determine 90-day Hip Society standardized complications after surgery.^19^ The 90-day Hip Society complications were the primary outcome measure of this study. Complications were defined according to Hip Society's definition (bleeding, wound complication, thromboembolic disease, neural deficit, vascular injury, dislocation/instability, periprosthetic fracture, abductor muscle disruption, deep periprosthetic joint infection, heterotopic ossification, bearing surface wear, osteolysis, implant loosening, cup-liner dissociation, implant fracture, revision surgery, revision, readmission, and death).^19^ Surgical complications were defined as complications excluding readmission for medical reasons (hyponatremia, GI bleed, atrial fibrillation, etc). Emergency department admissions were not recorded as readmissions unless the above listed complications were the reason for admission.

All statistical analyses were done with SPSS version 26 (IBM). Power analysis demonstrated that with 300 patients/group and an alpha of 0.05, there was 80% power to detect a 5% difference in complication rates between cohorts. Patient demographic and perioperative complications were compared between DA and PL groups, and the 50 case cohorts, using the Fisher exact test and chi-square test for categorical variables. Tests were deemed significant with a *P* value <0.05. Additional analyses were done comparing the first 50 with the last 50 cases for each surgeon.

## Results

There was a total of 600 patients, 300 patients within the DA group and 300 within the PL group. The DA group completed 100 cases within 11 to 16 months among the three surgeons, while the PL group completed 100 cases within 9.5 and 14 months among the three surgeons. When comparing the DA and PL cohorts, there were no significant differences in age, BMI, or indication (Table 1). A statistically significant difference was observed in sex between groups (60.0% women in the DA group versus 52.0% women in the PL group, *P* < 0.04).

**Table 1:** Patient Demographics in the PL Versus DA Cohorts

Demographics & Surgical Indications	DA	PL	*P* Value
Age (yr), mean ± SD	65.8	64.8	*P* = 0.27
Sex (% women)	181/300 (60.3%)	156/300 (52%)	*P* = 0.04
BMI (kg/m^2^)	30.2 ± 5.7	29.3 ± 6.1	*P* = 0.05
Surgical indications			
Osteoarthritis	260 (86%)	254 (84.5%)	*P* = 0.78
Fracture	30 (10%)	30 (10%)	
Osteonecrosis	10(3.3%)	14 (4.7%)	
Posttraumatic	0 (0%)	2 (0.6%)	

BMI = body mass index, DA = direct anterior, PL = posterolateral

### First 50 Case Cohort: Direct Anterior Versus Posterolateral

When comparing the DA versus PL surgeons' first 50 cases, there were no significant differences in revision surgeries (PL 4.0% vs. DA 1.0%, *P* = 0.15), surgical complications (PL 9.0% vs. DA 4.0%, *P* = 0.06), and total complications (PL 13.0% vs. DA 7.0%, *P* = 0.09) (Table 2).

**Table 2:** Complications in the First 50 Case Cohorts for PL Versus DA

Complications and Revisions	PL	DA	*P* Value
Revision surgeries, first 50	4% (6/150)	1% (2/150)	*P* = 0.15
Surgical complications, first 50	9% (14/150)	4% (6/150)	*P* = 0.06
Total complications, first 50	13% (20/150)	7% (11/150)	*P* = 0.09

DA = direct anterior, PL = posterolateral

Eight combined revision surgeries occurred in the first 50 case cohorts. Six revision surgeries occurred in the PL group (two for superficial wound I + D, two for periprosthetic fracture, one for aseptic loosening, and one for periprosthetic joint infection [PJI]) and two in the DA group (one periprosthetic fracture and one PJI).

Twenty surgical complications occurred in the first 50 case cohorts. Fourteen occurred in the PL group (four hip dislocations, one aseptic loosening, two superficial wound complications, three periprosthetic fractures, two PJIs, one venous thromboembolism/pulmonary embolism [VTE/PE] and one quadriceps tendon tear). Six complications occurred in the DA group (one hip dislocation, one superficial wound complication, one periprosthetic fracture, one PJI, and two VTE/PE).

Thirty-one total complications occurred in the first 50 case cohorts. Twenty occurred in the PL group (14 surgical complications, one readmission for chronic heart failure [CHF] exacerbation, two readmissions for gastrointestinal [GI] bleeds, one readmission for atrial fibrillation, one readmission for lower extremity cellulitis, and one readmission severe nausea/vomiting). Eleven total complications occurred in the DA group (six total surgical complications, one readmission for CHF exacerbation, one readmission for hyponatremia, one readmission for subdural hematoma, one readmission for atrial fibrillation, and one readmission for fatigue/fall.

### First 50 Versus Second 50 Case Cohorts

When analyzed separately, both the DA and PL cohorts had slightly lower rates of complications between the first 50 and second 50 case cohorts but neither were statistically significant (Table 3).

**Table 3:** First 50 Versus Second 50 Case Cohorts

Complications and Revisions	First 50	Second 50	*P* Value
DA			
Revision surgeries	1% (2/150)	0% (0/100)	*P* = 0.49 (FE)
Surgical complications	4% (6/150)	2% (3/150)	*P* = 0.31
Total complications	7% (11/150)	6% (9/150)	*P* = 0.64
PL			
Revision surgeries	4% (6/150)	2% (3/150)	*P* = 0.31
Surgical complications	9% (14/150)	4% (6/150)	*P* = 0.064
Total complications	13% (20/150)	6.5% (10/150)	*P* = 0.054

DA = direct anterior, PL = posterolateral

Three revision surgeries occurred in the second 50 case cohort. Three revision surgeries occurred in the PL cohort (three for PJI) and 0 revision surgeries in the DA cohort.

Nine surgical complications occurred in the second 50 case cohorts. Six surgical complications occurred in the PL group (two dislocations, one intraoperative fracture, and three PJI) and three surgical complications in the DA group (two dislocations and one intraoperative fracture).

Nineteen total complications occurred in the second 50 case cohorts. Ten total complications occurred in the PL group (six surgical complications, one readmission for bronchitis, one readmission for vertebral fracture, one readmission for sickle crisis, and one readmission for colitis). Nine total complications occurred in the DA group (three surgical complications, one readmission for Non-ST-Elevation Myocardial Infarction [NSTEMI] one readmission for pneumonia, one readmission for pyelonephritis, one readmission for fatigue/fall, and two readmissions for CHF exacerbation).

**Figure 1:**
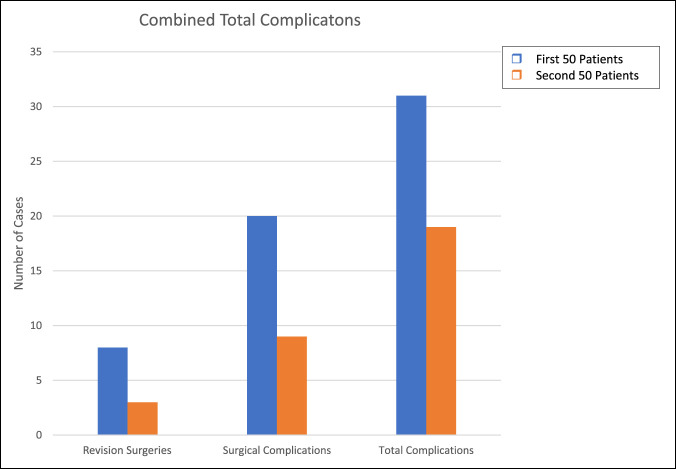
Graph showing combined total complications, surgical complications, and revision surgeries among both DA and PL for their combined first 50 cases versus their combined second 50 cases. DA = direct anterior, PL = posterolateral

For all surgeons combined, there were higher complication rates when comparing the first 50 cases with the second 50 cases for revision surgeries (2.8% vs. 1.2%, *P* = 0.2), surgical complications (6.6% vs. 3.0%, *P* = 0.036), and total complications (10.0% vs. 6.3%, *P* = 0.06) (Figure 1).

## Discussion

The DA approach for THA has increased in popularity over the past two decades for a variety of reasons including perceived benefits of decreased postoperative pain and improved early functional outcomes.^20,21^ With this increase in use, it is important to understand whether a clinical benefit outweighs the potential risk of adopting this procedure. A commonly cited drawback of the DA approach is the learning curve associated with performing the procedure. The current literature regarding the learning curve for the DA approach focuses primarily on the experiences of mid-career arthroplasty surgeons who switch from an alternative approach, such as PL, to the DA. There is little reported evidence regarding the experiences of arthroplasty fellowship-trained surgeons who routinely performed the DA approach with supervision during their training. To our knowledge, this is the first study to assess learning curves comparing recent fellowship-trained DA surgeons with recent fellowship-trained PL arthroplasty surgeons. Although there was a significant curve for all surgeons in the first 50 cases, we reported no differences in the learning curve regarding complications when comparing the DA and PL approach for fellowship-trained arthroplasty surgeons.

In contrast to other studies which have evaluated this learning curve in the context of experienced arthroplasty surgeons transitioning to DA, our study evaluated the learning curve among newly trained fellowship surgeons. Only two previous studies to our knowledge have looked at the learning curve of new surgeons.^22,23^ Burnham et al evaluated 286 patients between four different fellowship-trained arthroplasty surgeons and found a learning curve of approximately 40 to 50 cases when looking at medical and surgical complications.^22^ Garbarino et al examined outcomes in a surgeon with postfellowship specialty training THA. This study reported lower rates of complications of the DA approach compared with the existing literature.^22,23^ Our findings are in agreement with the above studies which suggest with appropriate training, there is no notable increase in complications with the DA versus other approaches. Of note, although we found no differences in learning curve among surgical approaches, we did report a notable decrease in revision surgeries, total and surgical complications when comparing the first 50 with last 50 cases with the combined surgical approach cohorts and within each approach group. In contrast to the previous studies, our study focused on comparing fellowship-trained surgeons during their first year of practice performing two different approaches. The lack of difference between the two groups may be explained by the inherent learning curve accompanied in all surgical trainees and junior attendings rather than a true steeper learning curve among the DA approach.^24,25^

Previous studies have cited increased rates of periprosthetic fracture with utilization of the DA approach.^26,27^ The potential increased risk of periprosthetic fractures using the DA approach may be attributed to the greater difficulty in femoral exposure and preparation, especially during the learning curve.^26^ Our overall rate of periprosthetic fracture was low in both the DA group (0.33%, n = 1) and the PL group (0.66%, n = 2), suggesting no increased risk for the DA approach. There is also concern given the difficult exposure, increasing BMI may lead to increasing periprosthetic fractures.^26,28^ Our findings did not support BMI as a risk factor, although there were limited number of fractures for comparison. The lone periprosthetic fracture in the DA group had a BMI of 27 and occurred during the first 50 cases. These results suggest that fellowship training in the DA approach may have a protective effect for reducing periprosthetic fracture risk. Nevertheless, these results should be considered carefully because we can only speculate on the exact mechanisms. The high-volume experience during a year of fellowship training offers ample opportunity to familiarize the surgeon with the nuances of hip arthroplasty including component positioning specific to each approach. This may confer potentially improved outcomes in patients because this has been demonstrated in the setting of femoral neck fractures treated with hemiarthroplasty.^29^ That being said, the role of volume and surgical outcomes is out of the scope of this study. We intended to identify the learning curve after fellowship between two different approaches, and we hypothesize that fellowship may play a role in bridging training to help not eliminate but merely flatten this learning curve.

Proponents of the DA approach also suggest a lower dislocation rate compared with the PL approach. Available literature for the DA approach provides a lower rate of dislocation with the rates ranging from 0% to 0.96%.^7,30^ Our study found overall low rates of dislocation. Six dislocations were observed in the PL group (2.0%) and three dislocations in the DA group (1.0%), with no statistically significant difference between groups. However, this study was likely underpowered to detect statistical differences in one low incidence surgical outcome measure, such as dislocations.

Previous studies have suggested that the anterior approach may be associated with higher rates of wound complications and have advised against using the anterior approach in obese patients because of the concern of overhanging abdomen predisposing to wound dehiscence.^8,31,32^ In our study, the rates of wound complications were 0.67% and 0.3% for PL and DA, respectively, despite a nonnotable higher BMI among the DA group. The PL superficial wound complication rate is similar to that reported by Purcell et al^32^ who reviewed 4651 patients undergoing THA. They noted a higher rate among the DA approach, 1.07%. The difference in our results may be due to the use of negative pressure incisional wound vacs in at-risk patients by some DA surgeons or once again related to fellowship training to reduce learning curve complication events.

Limitations of this study include the inherent weaknesses associated with the retrospective nature of this study. This was also a rather small sample size for each surgeon, but previous literature supports 40 to 50 cases for the DA learning curve, which is included in our sample size for each surgeon. Notably, these were truly the first 100 cases of our DA surgeons because there was no primary THA performed through another approach by these surgeons. This study also reports the results between six different surgeons with varying perioperative management, intraoperative techniques, and postoperative protocols at different institutions, which may bias results but does lend to the generalizability of this study. Despite this, the goal was to capture the learning curve regarding the complication rate of a newly trained fellowship surgeon in practice. Our study illustrated the importance of fellowship training and the humility and prudence one must exhibit during their first year of practice, regardless of the approach.

## Conclusions

There is an inevitable learning curve for all THA surgeons during their first 50 cases in practice. Despite perceptions for a steeper learning curve for DA THA, there were no differences in the learning curve when comparing the DA and PL approaches for fellowship-trained arthroplasty surgeons. With proper training, early-career surgeons can safely perform THA with similar complication rates, regardless of the approach.
